# Technical phosphoproteomic and bioinformatic tools useful in cancer research

**DOI:** 10.1186/2043-9113-1-26

**Published:** 2011-10-03

**Authors:** Elena López, Jan-Jaap Wesselink, Isabel López, Jesús Mendieta, Paulino Gómez-Puertas, Sarbelio Rodríguez Muñoz

**Affiliations:** 1 Centro de Investigación i+12 del Hospital Universitario 12 de Octubre, Avda de Córdoba s/n Madrid, 28041, Spain; 2 Centro de Biología Molecular "Severo Ochoa" (CSIC-UAM) Campus de Cantoblanco, c/Nicolás Cabrera, 1, 28049 Madrid, Spain; 3 Biomol-Informatics, S.L., Parque Científico de Madrid, Campus de Cantoblanco, c/Faraday 7, 28049 Madrid, Spain; 4 Servicio de Hematología Hospital QUIRÓN, Madrid, Diego de Velázquez 1 28223, Pozuelo Madrid Spain; 5 Servicio de Digestivo, Hospital Universitario 12 Octubre, Avda de Córdoba s/n Madrid, 28041, Spain

## Abstract

Reversible protein phosphorylation is one of the most important forms of cellular regulation. Thus, phosphoproteomic analysis of protein phosphorylation in cells is a powerful tool to evaluate cell functional status. The importance of protein kinase-regulated signal transduction pathways in human cancer has led to the development of drugs that inhibit protein kinases at the apex or intermediary levels of these pathways. Phosphoproteomic analysis of these signalling pathways will provide important insights for operation and connectivity of these pathways to facilitate identification of the best targets for cancer therapies. Enrichment of phosphorylated proteins or peptides from tissue or bodily fluid samples is required. The application of technologies such as phosphoenrichments, mass spectrometry (MS) coupled to bioinformatics tools is crucial for the identification and quantification of protein phosphorylation sites for advancing in such relevant clinical research. A combination of different phosphopeptide enrichments, quantitative techniques and bioinformatic tools is necessary to achieve good phospho-regulation data and good structural analysis of protein studies. The current and most useful proteomics and bioinformatics techniques will be explained with research examples. Our aim in this article is to be helpful for cancer research via detailing proteomics and bioinformatic tools.

## Introduction

Phosphoproteomics plays an important role in our understanding of how phosphorylation participates in translating distinct signals into the normal and or abnormal physiological responses, and has shifted research towards screening for potential therapies for diseases and in-depth analysis of phosphoproteomes. These issues can also be studied by structural analysis of proteins and bioinformatic tools. Specific domains discriminate between the phosphorylated *vs*. the non-phosphorylated state of proteins, based on the conformational changes induced by the presence of a negatively-charged phosphate group in the basal state of the phosphopeptide [1]

Phosphorylated proteins, chemically quite stable, are prone to enzymatic modification, so that when tissues or cells are lysed, it is very likely that further enzymatic reactions will occur [2]. Good sample preparation is the key to successful analysis. These will generally be snap-frozen and treated with phosphatase inhibitors to avoid modifying phosphopeptides during sample work-up [3,4]. Also, it is critical to avoid salts and detergents, which can decrease the recovery of phosphopeptides or interfere with subsequent analysis [5]. Phosphopeptides generally make up a small portion of the peptides in a given protein sample, making detection difficult. Their enrichment [*e.g*. via Immobilised metal ion affinity chromatography (IMAC), Titanium dioxide metal-based chromatography (TiO_2_), Zirconium dioxide (ZrO_2_), Sequential elution from IMAC (SIMAC) or Calcium phosphate precipitation] helps to combat this problem.

When combining the previously mentioned phosphoenrichments with Strong cation and anion exchange (SCX and SAX) or Hydrophilic interaction chromatography (HILIC), large-scale phosphoproteomic studies of interest can be carried out successfully [6]. If the goal of the research study includes quantification of phosphorylated proteins, there are several useful techniques [*e.g*. Stable Isotope Labelling with Amino acids in cell Culture (SILAC), Isobaric Tag for Relative and Absolute (iTRAQ), Absolute Quantitation (AQUA), Multiple Reaction Monitoring (MRM), or Label-free quantification], which allow important large-scale phosphoproteomic studies [7-19]

**Figure 1:**
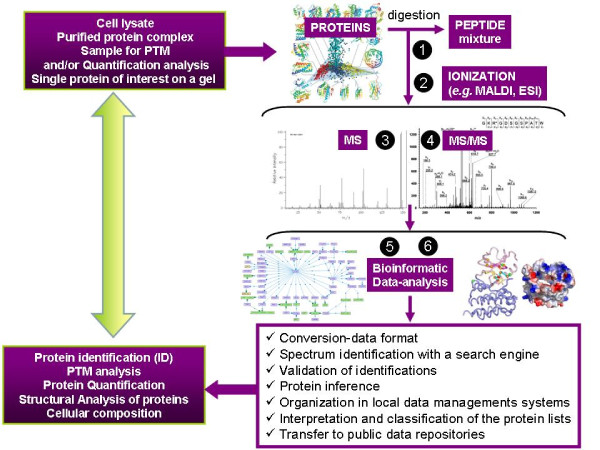
**A prototypical proteomics pipe-line coupled to bioinformatics useful for clinical research**. Depending on the application, different samples processed and fed into the proteomics pipeline yield different results. The pipeline's several steps are listed in the different panels: (1) proteolytic digest, (2) the separation and ionization of peptides, (3) their analysis by mass spectrometry, (4) fragmentation of selected peptides and analysis of the resulting MS/MS spectra and, (5) (6) data-computer bioinformatic-analysis, which mainly includes: Conversion-data format, Spectrum identification with a search engine, Validation of identifications, Protein inference, Organization in local data managements systems, Interpretation and classification of the protein lists, Transfer to public data repositories, Identification and Classification of proteins, Quantification, Structural Analysis of proteins, PTM analysis and Cellular composition.

Once the phosphorylation state of a protein, constitutive or associated to cancer disorders has been established by proteomics methods, a range of bioinformatics methods permits deeper study of its properties and contacts. Using sequence analysis, sequence comparison, virtual approaches of protein-protein, protein-ligand interaction or molecular dynamics simulations, initial physical information can be applied for the potential development of personalized approaches, aimed at the concept of personalized medicine. Bioinformatics covers a wide spectrum of techniques for the generation and use of beneficial information from structure, sequence or relationships among biological items (DNA, RNA, proteins, macromolecular complexes, etc) [20,21]. From all these methods, those most useful in clinical cancer studies are: Ascore, PhosphoScore, data analysis from Next-Generation Sequencing, studies of sequence comparison and sequence--structure relationship, homology modelling and the more sophisticated rational drug design and molecular dynamics techniques. Using phosphoproteomics together with structural analysis of proteins and bioinformatic tools, important biological understanding of malignant diseases can be achieved. A prototypical proteomics coupled to bioinformatics pipe-line useful for clinical cancer research is illustrated (Figure 1)

### Current MS-based resins to isolate phosphoproteins-phosphopeptides useful for cancer research

#### Immobilised metal ion affinity chromatography (IMAC), Titanium dioxide metal-based chromatography (TiO_2_), Sequential elution from IMAC (SIMAC) and Zirconium dioxide (ZrO_2_)

TiO_2 _and IMAC are capable of binding negatively charged phosphate groups from aqueous solutions. Simple and complex samples containing phosphopeptides and non-phosphorylated peptides are dissolved in an acidic solution to reduce the non-specific binding of acidic peptides (*e.g*. those containing aspartic acid and glutamic acid), and to stimulate the electrostatic interactions between the negatively charged peptides, mainly phosphopeptides, and the metal ions. The phosphopeptides isolated are eluted from the stationary phase using alkaline buffers [22]

Both resins (TiO_2 _and IMAC) have the drawback of binding acidic non-phosphorylated peptides (negatively charged peptides). Peptides containing acidic amino acid residues, glutamic acid and aspartic acid, can also bind to the metal ions. Ficarro *et al *(2002) circumvented this difficulty with IMAC (Fe^3+^) by converting acidic amino acid residues to methyl esters [23-29]. Heck *et al *[27] suggested esterification of the acidic residues prior to the MS analysis, as they observed a number of non-phosphorylated peptides in their analysis. Larsen *et al *[34] achieved higher specificity and yield compared to IMAC (Fe^3+^) for the selective enrichment of phosphorylated peptides from model proteins when using 2,5-dihydroxybenzoic acid (DHB) with TiO_2_. In addition, more phosphopeptides are bound to the metal ions and more phosphopeptides can be eluted by using ammonium hydroxide as the eluent by use of glycolic acid in the loading buffer of TiO_2 _[30-35]

SIMAC allows enrichment of mono and multiply-phosphopeptides in a single experiment, and, from complex biological samples. Mono-phosphorylated peptides mainly elute from IMAC (Fe^3+^) under acidic conditions whereas multi-phosphorylated peptides elute at high basic pH. Following SIMAC protocol, TiO_2 _allows capture of the unbound mono-phosphorylated peptides in the combined IMAC flow-through and washing steps [35,36]

ZrO_2_, like the phosphoenrichments previously mentioned, is very useful for phosphopeptide isolation prior to MS analysis. The strong affinity of ZrO_2 _nanoparticles to phosphopeptides enables the specific enrichment of phosphopeptides from a complex peptide mixture in which the abundance of phosphopeptides is two orders of magnitude lower than that of nonphosphopeptides [37,38]

#### Calcium phosphate precipitation (CPP), Strong cation and anion exchange (SCX and SAX) and Hydrophilic interaction chromatography (HILIC)

CPP consists of a pre-fractionation step in order to simplify and enrich phosphopeptides from complex samples. CPP coupled to two step IMAC (Fe^3+^) procedure resulted in the observation of a higher number of phosphopeptides recovered. Phosphopeptides are precipitated by adding 0.5 M NaHPO_4 _and 2 M NH_3_OH to the peptide-mixture followed by 2 M CaCl_2_. The washed pellet (with 80 mM CaCl_2_) is dissolved in 5% of formic acid. Before isolating the phosphopeptides by IMAC (Fe^3+^), the resulting peptide-mixture is desalted via reversed phase chromatography (RP) [39]

A positively charged analyte is attracted to a negatively charged solid-support, and a negatively charged analyte is attracted to a positively charged solid-support during SCX and SAX operations respectively. SCX and SAX has been successfully combined with IMAC and resulted in greater recovery and identification by MS of interesting phosphorylated peptides originating from yeast pheromone signalling pathway and membrane proteins respectively [28,40]

HILIC consist of a liquid/liquid extraction system between the mobile and stationary phase. A water-rich layer on the surface of the stationary phase (polar) is formed; therefore a distribution of the analytes between these two layers will occur. Weak electrostatic mechanisms as well as hydrogen donor interactions between neutral polar molecules under high organic elution conditions occur during HILIC operations. Moreover, more polar compounds have stronger interaction with the stationary aqueous layer than less polar compounds, resulting in a stronger retention [41]

#### Pros and Cons of Phosphoproteomic tools

Using IMAC, TiO_2 _and ZrO_2_, the negatively charged phosphopeptides are purified by their affinity to positively charged metal ions. However, some of these methods experience the problem of binding acidic, non-phosphorylated peptides. Ficarro *et al *[29] bypassed this problem on IMAC (Fe^3+^) by converting acidic peptides to methyl esters but increased the spectra complexity and required lyophilization of the sample, causing adsorptive losses of phosphopeptides in particular. TiO_2 _chromatography using DHB was introduced as a promising strategy by Larsen *et al *[34]. TiO_2_/DHB resulted in higher specificity and yield compared to IMAC (Fe^3+^) for the selective enrichment of phosphorylated peptides from model proteins (*e.g*. lactoglobulin bovine, casein bovine). TiO_2 _offers increased capacity compared to IMAC resins in order to bind and elute mono-phosphorylated peptides. TiO_2 _exploits the same principle as IMAC, and is similarly prone to nonspecific retention of acidic nonphosphorylated peptides. However, when loading peptides in DHB, glycolic and phthalic acids, nonspecific binding to TiO_2 _is reduced, thereby improving phosphopeptide enrichment without chemical modification of the sample. SIMAC appeared as a phosphopeptide enrichment tool which exploits the properties of IMAC coupled to TiO_2_, thus facilitating more refined studies [36]

Another phosphopeptide enrichment prior to mass spectrometric analysis is ZrO_2 _[37] and its principle is based on metal affinity chromatography like IMAC and TiO_2_. ZrO_2 _permits the isolation of single phosphorylated peptides in a more selective manner than TiO_2 _[30]

Strategies which consist of fractionating and subsequently enriching phosphopeptides on a proteome wide scale are based on SCX/SAX and HILIC interaction chromatography. Calcium phosphate precipitation is also a useful pre-fractionation step to simplify and enrich phosphopeptides from complex samples which can be coupled to IMAC and TiO_2 _[13]. Mainly those phosphopeptides from highly expressed proteins within cells can be purified, while those from phosphorylated proteins with low level expression (*e.g*. kinases) do not bind so well to those resins. This is an important limitation concerning phosphoenrichment methods and is due to the low proportion of this kind of protein, or, their available amount binds to metal ions although not sufficiently so as to be detected by MS.

The combination of SCX with IMAC has been proven, resulting in a huge number of phosphorylated residues identified (over 700 including Fus3p kinase). Although more than 100 signalling proteins and functional phosphorylation sites, including receptors, kinases and transcription factors, have been identified, it is clear that only a fraction of the phosphoproteome has been revealed [7,40]

Combinations of HILIC with IMAC have been proven in clinical studies (*e.g*. HeLa samples), with the result of the identification of a large number of phosphorylated residues (around 1000) [41]

Improvement in methodologies to enrich for phosphorylated residues from kinases is clearly necessary. However, this is not straightforward for several reasons: the low abundance of those signalling molecules within cells, the stress/stimulation time-duration, as only a small fraction of phosphorylated kinases are available at any given time as a result of a stimulus and the time adaptation over signalling pathways [5]

### Current phosphoproteomic MS-based quantitative strategies presently used for cancer research

#### Stable Isotope Labelling with Amino acids in cell Culture (SILAC), Isobaric Tag for Relative and Absolute (iTRAQ), Absolute Quantitation (AQUA), Multiple Reaction Monitoring (MRM) and ^18^O labelling

SILAC is a technique based on MS that detects differences in protein abundance among samples using non-radioactive isotopic labelling. Two populations of cells are cultivated in cell culture. One of the cell populations is fed with growth medium containing normal amino acids. The second population is fed with growth medium containing amino acids labelled with stable (non-radioactive) heavy isotopes. For example, the medium can contain arginine labelled with six carbon-13 atoms (^13^C) instead of the normal carbon-12 (^12^C). When the cells are growing in this medium, they incorporate the heavy arginine into all of their proteins. All of the arginine containing peptides are now 6 Da heavier than their normal counterparts. The trick is that the proteins from both cell populations can be combined and analyzed together by MS. Pairs of chemically identical peptides of different stable-isotope composition can be differentiated via MS owing to their mass difference [42-45]

iTRAQ uses isotope-coded covalent tags and is based on the covalent labelling of the N-terminus and side chain amines of peptides from protein digestions with tags of varying mass. There are currently two mainly used reagents: 4-plex and 8-plex, which can be used to label all peptides from different samples/treatments. These samples are then pooled and usually fractionated by nano liquid chromatography and analyzed by tandem MS (MS/MS). The fragmentation of the attached tag generates a low molecular mass reporter ion that can be used to relatively quantify the peptides and the proteins from which they originated. The signals of the reporter ions of each MS/MS spectrum allow for calculating the relative abundance (ratio) of the peptide(s) identified by this spectrum. In contrast to SILAC and AQUA (described below), it is during MS/MS experiments, that relative quantification of peptides takes place [46-50]

AQUA was developed for the precise determination of protein expression and post-translational modification (PTM) levels. A peptide from a protein is constructed synthetically containing stable isotopes, and the AQUA peptide is the isotopically labelled synthetic peptide. The synthetic peptides can be synthesized with PTMs. The stable isotopes are incorporated into the AQUA peptide by using isotopically "heavy" amino acids during the synthesis process of the peptide of interest (native peptide). The synthetic peptide has a mass increase of *e.g*. 10Daltons, due to the incorporation of a ^13^C_6 _and ^15^N_4_- arginine into the synthetic peptide, compared to the native peptide. The mass difference between the native and the synthetic peptide allows the mass spectrometer to differentiate between the two forms - both forms have the same chemical properties - resulting in the same chromatographic retention, ionization efficiency, and fragmentation distribution [51-53]

MRM requires that knowledge of the sequence of the protein be known in order to calculate precursor and fragment ion values, which can be used to trigger dependent ion scans in a qTRAP (hybrid triple quadrupole linear ion trap mass spectrometer). It can also be used to perform a precursor ion and neutral loss scan, to identify unknown phosphopeptides from a complex mixture, and is a powerful method for the identification and quantification of PTMs in proteins. Indeed, MRM has been used by White *et al *to identify and quantify tyrosine phosphorylated kinases for hundreds of nodes within a signalling network and across multiple experimental conditions. White *et al.; *Cox *et al.*, and other relevant scientists [48,49,54,55] applied this strategy for phospho quantitative analysis of signalling networks, identifying and quantifying a high number of tyrosine phosphorylated peptides, obtaining an extremely high percentage of signalling nodes covered.

^18^O labelling is a label-free strategy that incorporates a stable isotope ^18^O-labelled ″universal″ reference sample as a comprehensive set of internal standards for analyzing large sample sets quantitatively. As a pooled sample, the ^18^O-labelled ″universal″ reference sample is spiked into each individually processed unlabelled biological sample and the peptide/protein abundances are quantified based on ^16^O/^18^O isotopic peptide pair abundance ratios that compare each unlabelled sample to the identical reference sample. This approach also allows for the direct application of label-free quantitation across the sample set simultaneously along with the labelling-approach (*e.g*., dual-quantitation) since each biological sample is unlabelled except for the labelled reference sample that is used as internal standard. The effectiveness of this approach for large-scale quantitative proteomics has been demonstrated by Qian *et al *2009; Wong *et al *2008 and other important scientists, giving relevant clues for malignant diseases [56,57]

### Some examples of phosphorylated proteins involved in relevant clinical diseases explaining how useful phosphoproteomic tools are for those clinical investigations

Some drugs that bind to microtubules and block mitosis are ineffective in cancer treatment; others show inexplicable focal efficacy. The vinca alkaloids are useful for treating lymphoma, neuroblastoma and nephroblastomas, whereas taxol is useful for advanced breast cancer and ovarian cancer. It is not known why these drugs are not all equally effective nor is it known why they have different therapeutic value against different cancers. Steen *et al *[58] examined the role of phosphorylation on the dynamics of the anaphase promoting complex (APC), observing distinct phosphorylation states of the APC in response to different antimitotic drugs and suggest that they may explain some of these differences. Cells from different tissues or with different mutations, or cells under different physiological stresses such as hypoxia, may differ in their response to spindle poisons and would reflect those differences in different sites of phosphorylation.

Differences in spindle checkpoint phosphorylation may reveal new features of the mitotic state. The ability to characterise drug candidates based on the spectrum of APC phosphorylations may facilitate the discrimination of the response of tumours to drugs and the identification of new means of checkpoint control.

The authors suggested that the results of their study indicate that the term mitotic arrest is a misnomer: arrest is a dynamic state in which some cells enter apoptosis and other cells revert to interphase. The ability to observe biochemical events during arrest could be very important for understanding antiproliferative treatments.

Exploring the dynamics of phosphorylation makes great demands on the accuracy of quantitation. Most MS-based quantitative approaches including SILAC and iTRAQ give relative data, meaning that one state of phosphorylation is determined relative to another phosphorylation state. These data can help to establish the kinetics of a pathway. These approaches allowed the measurement of specific quantitative changes in APC phosphorylation in cells arrested in nocodazole for varying periods. If these dynamics can be correlated with the process by which the arrested state is resolved, they may provide us with new tools to understand the mitotic process and to find more effective drug targets in cancer [59-61]

Development of drugs for specific biological pathways with increased specificity and reduced toxicity has validated the long-held belief in the cancer research community that a precise molecular understanding of cancer can result in cancer therapy.

An example of cancer-specific drugs is the development of Herceptin - a monoclonal antibody against the HER2 receptor for breast cancer therapy. HER2 is an important target in cancer. HER2 overexpression increases tumour cell proliferation, invasiveness and predicts poor prognosis. Wolf-Yadlin and other scientists [48,49,58-61] have used phosphoproteomics and MS to investigate the role of phosphorylation in the effects of HER2 overexpression on EGF- and HRG-mediated signalling of erbB receptors. They identified specific combinations of phosphorylation sites that correlate with cell proliferation and migration and that potentially represent targets for therapeutic intervention. 68 out of 322 phosphorylation sites could be analysed kinetically and it marks an important breakthrough in the characterisation of the erbB receptor signalling network in tumours and illustrates the importance of understanding protein phosphorylation.

Mitochondria play a central role in energy metabolism and cellular survival and consequently mitochondrial dysfunction is associated with a number of human pathologies. Mitochondrial dysfunction is linked to insulin resistance in humans with obesity and type 2 diabetes. Zhao *et al *(2011) [62] studied the phosphoproteome of the mitochondria isolated from human skeletal muscle. They revealed extensive phosphorylation of inner membrane protein complexes and enzymes combining TiO_2 _with reverse phase chromatography coupled to MS analysis. 155 distinct phosphorylation sites in 77 mitochondrial phosphoproteins including 116 phosphoserine, 23 phosphothreonine and 16 phosphotyrosine residues were identified. They also assigned phosphorylation sites in mitochondrial proteins involved in amino acid degradation, importers and transporters, calcium homeostasis and apoptosis. Many of these mitochondrial phosphoproteins are substrates for protein kinase A, protein kinase C, casein kinase II and DNA-dependent protein kinase. The high number of phosphotyrosine residues suggests an important role for tyrosine phosphorylation in mitochondrial signalling. Many of the mitochondrial phosphoproteins are involved in oxidative phosphorylation, tricarboxylic acid cycle and lipid metabolism *e.g*. processes proposed to be involved in insulin resistance [63].

In this study [64] the most prevalent form of cellular protein post-translational modifications (PTMs) reversible phosphorylation is emerging as a central mechanism in the regulation of mitochondrial functions [64-71]. Boja *et al *(2009) [50] successfully monitored phosphorylation sites of mitochondrial proteins including adenine nucleotide translocase, malate dehydrogenase and mitochondrial creatine kinase. Among them, four proteins exhibited phosphorylation changes with these physiological stimuli: BCKDH-E1α subunit increased phosphorylation at Ser337 with DCA and de-energization, apoptosis-inducing factor phosphorylation was elevated at Ser345 with calcium, ATP synthase F1 complex α subunit and mitofilin dephosphorylated at Ser65 and Ser264 upon de-energization. This screening validated the iTRAQ technology as a method for functional quantitation of mitochondrial protein phosphorylation as well as providing insights into the regulation of mitochondria via phosphorylation [69-71]

White *et al *[48,49] applied iTRAQ and MRM for phosphor-quantitative analysis of signalling networks identifying and quantifying 222 tyrosine phosphorylated peptides, obtaining an extremely high percentage of signalling nodes covered. Ziwei Yu *et al *(2007) using AQUA as a novel system of *in situ *quantitative protein analysis, studied the protein expression levels of phosphorylated Akt (p-Akt). Activation of Akt in tumours is mediated via several mechanisms including activation of cell membrane receptor tyrosine kinases such as EGFR and loss of phosphatase PTEN with dephosphorylation of phosphoinositol triphosphate. Ziwei *et al *discovered that Akt activation in oropharyngeal squamous cell carcinoma (OSCC) is associated with adverse patient outcome, indicating that Akt is a promising molecular target in oropharyngeal squamous cell carcinoma [53]

White *et al *[59,61] defined the mechanisms by which EGFRvIII protein alters cell physiology, as it is one of the most commonly mutated proteins in GBM and has been linked to radiation and chemotherapeutic resistance. They performed a phosphoproteomic analysis of EGFRvIII signalling networks in GBM cells. They provided important insights into the biology of this mutated receptor including oncogene dose effects and differential utilization of signalling pathways. Clustering of the phosphoproteomic data set revealed a previously undescribed crosstalk between EGFRvIII and the c-Met receptor. They observed that treatment of the cells with a combination employing both EGFR and c-Met kinase inhibitors dramatically decreased cell viability *in vitro*.

Hoffert *et al *[72] carried out quantitative phosphoproteomic analysis of vasopressin-sensitive renal cells of rat inner medullary collecting duct cells by using IMAC and phosphorylation-site identification by MS combining label-free quantitation.

They identified 714 phosphorylation sites on 223 unique phosphoproteins from inner medullary collecting duct samples treated short term with either calyculin A or vasopressin. Rinschen *et al *[73] studied vasopressin's actionin renal cells related to the fact that the regulation of water transport depends on protein phosphorylation. Using SILAC with two treatment groups (0.1 nM dDAVP or vehicle for 30 min), they carried out quantification of 2884 phosphopeptides. The majority of quantified phosphopeptides did not change in abundance in response to dDAVP. Analysis of the 273 phosphopeptides increased by dDAVP showed a predominance of so-called "basophilic" motifs consistent with activation of kinases of the AGC family. Increases in phosphorylation of several known protein kinase A targets were found. Increased phosphorylation of targets of the calmodulin-dependent kinase family was also seen, including autophosphorylation of calmodulin-dependent kinase 2 at T286. Analysis of the 254 phosphopeptides decreased in abundance by dDAVP showed a predominance of so called "proline-directed" motifs, consistent with down-regulation of mitogen-activated or cyclin-dependent kinases. dDAVP decreased phosphorylation of both JNK1/2 (T183/Y185) and ERK1/2 (T183/Y185; T203/Y205), consistent with a decrease in activation of these proline-directed kinases in response to dDAVP.

Both ERK and JNK were able to phosphorylate residue S261 of aquaporin-2 in vitro, a site showing a decrease in phosphorylation in response to dDAVP in vivo. Their data support roles for multiple vasopressin V2-receptor-dependent signalling pathways in the vasopressin signalling network of collecting duct cells, involving several kinases not generally accepted to regulate collecting duct function. We should remark that Hoffert and co-workers carried out a very interesting research study, via a label-free quantitation strategy that measures phosphopeptide precursor ion abundances from extracted ion chromatograms (XIC).

The comparison of cellular phosphorylation levels for control, epidermal growth factor stimulus and growth factor combined with kinase inhibitors has been studied by Mann *et al *[74] using triple labelling SILAC coupled to SCX and TiO_2_.

They evaluated the effects of kinase inhibitors on the entire cell signalling network. From thousands of phosphopeptides, less than 10% had a response pattern indicative of targets of U0126 and SB202190, two widely used MAPK inhibitors. They found that the 83% of the growth factor-induced phosphorylation events were affected by either or both inhibitors, showing quantitatively that early signalling processes are predominantly transmitted through the MAPK cascades. In contrast to MAPK inhibitors, dasatinib, a clinical drug directed against BCR-ABL, which is the cause of chronic myelogenous leukemia, affected nearly 1,000 phosphopeptides. Their assay is streamlined and could become a useful tool in kinase drug development.

Knowlton *et al *[45] conducted quantitative mass spectrometry via SILAC and immunoaffinity purification of tyrosine phosphorylated peptides to profile candidate SRC-substrates induced by the CSF-1R tyrosine kinase by comparing the phosphotyrosine-containing peptides from cells expressing either CSF-1R or a mutant form of this RTK that is unable to bind to SFKs.

They identified uncharacterized changes in tyrosine phosphorylation induced by CSF-1R in mammary epithelial cells as well as a set of candidate substrates dependent on SRC recruitment to CSF-1R. Many of these candidates may be direct SRC targets as the amino acids flanking the phosphorylation sites in these proteins are similar to known SRC kinase phosphorylation motifs. Their collection of substrates includes proteins involved in multiple cellular processes including cell-cell adhesion, endocytosis and signal transduction. Analyses of phosphoproteomic data from breast and lung cancer patient samples identified a subset of the SRC-dependent phosphorylation sites as being strongly correlated with SRC activation, which represent candidate markers of SRC activation downstream of receptor tyrosine kinases in human tumours.

Integrins interact with extracellular matrix (ECM) and deliver intracellular signalling for cell proliferation, survival and motility. During tumour metastasis, integrin-mediated cell adhesion and migration on the ECM proteins are required for cancer cell survival and adaptation to the new microenvironment.

Chen Y *et al *[75] using SILAC, IMAC and MS profiled the phosphoproteomic changes induced by the interactions of cell integrins with type I collagen, the most common ECM substratum. The authors depicted an integrin-modulated phosphorylation network during cell-ECM protein interactions and revealed novel regulators for cell adhesion and migration, discovering that integrin-ECM interactions modulate phosphorylation of 517 serine, threonine or tyrosine residues in 513 peptides, corresponding to 357 proteins. Among these proteins, 33 key signalling mediators with kinase or phosphatase activity were subjected to siRNA-based functional screening. In their study, three integrin-regulated kinases, DBF4, PAK2 and GRK6, were identified for their critical role in cell adhesion and migration possibly through their regulation of actin cytoskeleton arrangement.

### Current Bioinformatics Tools useful for Phosphoproteomic Research in Cancer studies

#### PhosphoScore

Correct phosphorylation site assignment is a critical aspect of phosphoproteomic analysis. Large-scale phosphopeptide data sets that are generated through liquid chromatography-coupled tandem MS often contain hundreds or thousands of phosphorylation sites that require validation.

PhosphoScore is an open-source assignment program that is compatible with phosphopeptide data from multiple MS levels (MSn). It consists of an algorithm which takes into account the match quality and the normalized intensity of observed spectral peaks compared to a theoretical spectrum. It has been demonstrated by Ruttenberg *et al *[76] that PhosphoScore produces > 95% correct MS2 assignments from known synthetic data, > 98% agreement with an established MS2 assignment algorithm (Ascore), and > 92% agreement with visual inspection of MS3 and MS4 spectra. It was successfully used for the isolation of phosphopeptides from rat liver. The resulting phosphopeptides were enriched via IMAC and analized by MS allowing important data of phosphorylated proteins from rat liver.

#### Ascore

Ascore consists of a statistical algorithm that measures the probability of correct phosphorylation site localization based on the presence and intensity of site-determining ions in MS2 spectra. Phosphorylation sites with an Ascore ≥ 19 (corresponding to > 99% certainty) are usually considered unambiguously assigned. The Ascore algorithm is compatible with MS2 spectra and phosphorylation sites from phosphopeptides found only at the MS3 level are assigned by manual examination of the spectra (http://ascore.med.harvard.edu/ascore.php). To distinguish the correct site(s) of phosphorylation for each phosphopeptide, automated site assignment is performed on MS2 data using the Ascore algorithm. It was used for an interesting research study of the phosphoprotein aquaporin-2 (AQP2) that was also quantified. This particular AQP2 peptide was identified from an MS3 spectrum and contained three unambiguously assigned phosphorylation sites: Ser-256, Ser-261, and Ser-264. A previous phosphoproteomic study by the same group included MS-based quantification of AQP2 at Ser-256 and Ser-261. The dramatic increase in abundance of this phosphopeptide in vasopressin-treated samples was consistent with increased phosphorylation of AQP2 at Ser-256 in response to vasopressin [77]

#### Next Generation Sequencing

Next Generation Sequencing (NGS) has been recently used in a detailed study of genes involved in Colorectal Cancer (CRC) [78]. As a main conclusion of the study, the authors stated that sequencing of whole tumour exomes allowed prediction of the microsatellite status of CGC, facilitating, at the same time, the putative finding of relevant mutations. In addition, NGS can be applied to formalin-fixed and paraffin embedded material, allowing the renewed study of all the ancient material stored in the pathology departments [79].

#### Sequence-to-sequence and sequence-to-structure comparisons (MSA: multiple sequence analysis)

Once mutations or phosphorylation of modified residues have been found in sequencing or proteomics studies, routine sequence-to-sequence and sequence-to-structure comparisons (MSA: multiple sequence analysis) are applied to obtain valuable information on the nature of the functional implications of the mutated residues in the protein context. Multiple alignments of proteins, and mainly those based on the comparison of experimentally obtained-three dimensional atomic structures (structural alignments), are a very valuable source of information related to the evolutionary strategies followed by the different members of a family of proteins to conserve or modify their functions and structures [80]

The analysis of structural alignments allows the detection of at least three types of regions or multiple alignment positions according to conservation:

1. Conserved positions, usually key for function or structure maintenance.

2. Tree-determinant residues, conserved only in protein subfamilies and related to family-specific active sites, substrate binding sites or protein-protein interaction surfaces. These sites contain essential information for the design of family-specific activator or inhibitor drugs [81].

3. Positions that correspond to compensatory mutations that stabilize the mutations in one protein with changes in the other (correlated mutations). These sites are very effective for the detection of protein-protein interaction contacts [82], as they allow for the selection of the correct structural arrangement of two proteins based on the accumulation of signals in the proximity of interacting surfaces.

#### Homology modelling methods

As a consequence of the sequence-to-structure comparison, and in absence of experimental crystal structures, the homology modelling methods, can develop a 3D model from a protein sequence based on the structures of a crystallized homologous protein. The method can only be applied to proteins having a common evolutionary origin, as only for proteins that are hypothesized to be homologous, this assertion implies that their three-dimensional structures are conserved to a greater extent than their primary structures. For cases where a good homology hypothesis cannot be supported, alternative methods can be applied in order to obtain a putative 3D structure. These procedures, known as "far-homology modelling" or "threading" methods, provide structures with lower confidence compared to those generated using homology modelling methods.

**Figure 2:**
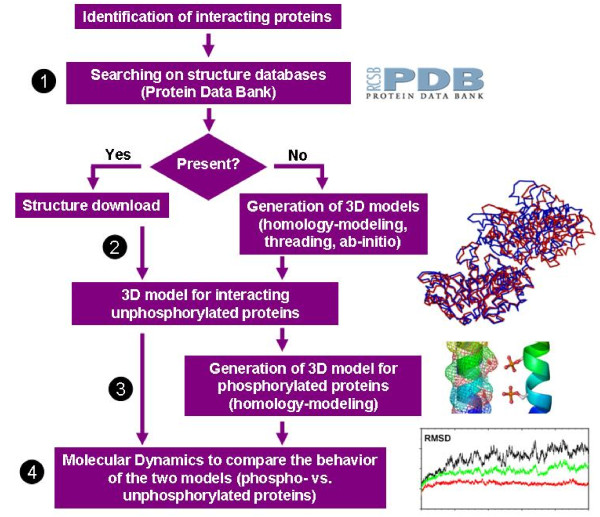
**Routine pipe-line for structural bioinformatics analysis of protein phosphorylated states**. Once the protein is identified, a sequence-based search (1) in the Protein Data Bank (http://www.rcsb.org/pdb) structure database is done to download a 3D structure suitable to be used in computational simulation studies. In the case that the protein is not present in the database, bioinformatics modelling methods are used to generate an approximate model of the desired structures (2). Next step consists of the generation of the 3D model for the single protein or the interacting pair of proteins both in the unphosphorylated (basal) or the phosphorylated states (3). Finally, a Molecular Dynamics approach is used to compare the behaviour of the two states. RMSD (root mean square distance) values are collected for several nanoseconds in order to obtain a quantitative measure of the differences (4).

Routine pipe-line for structural bioinformatics techniques used from structure identification to Molecular Dynamics analysis of the phosphorylated forms is summarized in Figure 2.

#### The 3D structure of the active centre of a protein of interest

Information on the 3D structure of the active centre of a protein of interest and/or its natural ligands can be used as a basis for the design of effective drugs. This rational drug design is usually performed using multiple docking experiments in the active centre of the said protein, requiring the use of advanced software such as Autodock-4 [83], that allows the evaluation of not only the docking to a rigid model of the active centre, but also a certain mobility of the side chain of enzyme residues to the ligand shape. Typically, all the calculated binding conformations to the target protein obtained in every docking run are clustered according to scoring criteria (as "lowest binding energy model" or "lowest energy model representative of the most-populated cluster") and sorted according to their estimated free energy of binding. These computer procedures are a useful cost-reducing tool to prospect and model new molecules with potential inhibiting properties or even successful future drugs. Recently, rational drug design approach has been used in the case of putative cancer therapies, focused on the pharmacological reactivation of mutant p53 [84]. This promising strategy implies the simultaneous use of several approaches for the identification of small molecules that target mutant p53, including "de novo" design and screening of chemical libraries.

#### Molecular dynamics (MD) techniques

Finally, molecular dynamics (MD) techniques are commonly used to obtain refined models for protein structure, protein-protein and protein-ligand interactions.

Molecular dynamics is a computational simulation technique in which atoms within molecules are allowed to interact for a period of time according to the principles of physics. In the case of proteins, the relevant forces taken into account are the electrostatic interactions (attractive or repulsive), Van der Waals interactions, and the properties of the covalent bond (length, angle, and dihedral angle). In general, simulation times for macromolecular protein complexes are up to 20 ns and the number of atoms of the simulated systems is in the order of up to 250,000, including solvent molecules. MD techniques have been used to simulate the individual behaviour of small protein or peptides [85], protein-protein interfaces and ligand-protein relationship in catalytic macromolecular complexes with GTPase activity [86,87] or kinases involved in cell signalling pathways (*e.g*. Src tyrosine kinase [88] or protein kinase B/Akt [89])

**Figure 3:**
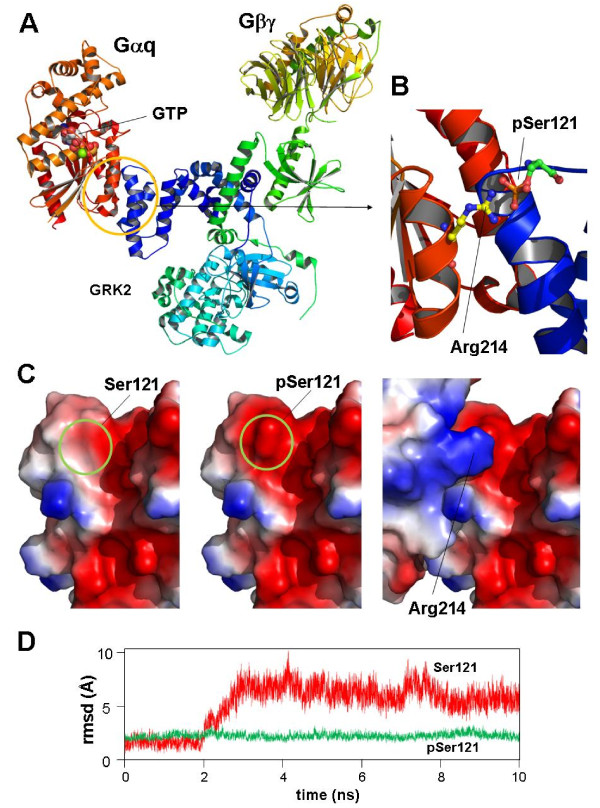
**Case study. Analysis of the structural interactions of GRK2 [Swiss-Prot: **P21146**], Gαq [Swiss-Prot: **P21279**] and Gβγ proteins [Swiss-Prot: **P62871**and Swiss-Prot: **P63212**] according to the crystallized structure of the macromolecular complex [PDB: **2BCJ**]**. A. Crystallized structure of the complex of GRK2, Gαq and Gβγ polypeptides. Position of a GTP molecule in Gαq active centre is indicated. B. Computer model of the electrostatic interaction between a putative phosphorylated GRK2-Ser121 residue and Arg214 of Gαq. C: Surface models for GRK2 protein in the vicinity of Ser121 residue. Left: Unphosphorylated Ser121; centre: model for the putative phosphorylated state of Ser121. Right: complementarity between the positively Arg214 and negative pSer121charged residues patched in both protein surfaces, probably implicated in the stabilization of the complex. D. Root mean square deviation (RMSD) plots of the protein domains implicated in the GRK2-Gαq interaction in presence (green) or absence (red) of phosphorylated Ser121 during a simulation of molecular dynamics. Plots are presented solely to illustrate the putative stabilization of the complex after Ser121 phosphorylation. Figure plots were generated using PyMOL Molecular Graphics System, Schrödinger, LLC.

Figure 3 shows, as an example, the bioinformatics analysis of the crystallized macromolecular complex of activated G proteins [90], composed of, Gαq and Gβγ proteins. GRK2 has been implied in the inhibition of WNT signalling [91], a pathway that plays a central role in the etiology of colorectal cancer. GRK2 plays a pivotal role in the G protein-coupled receptor (GPCR) desensitization and re-sensitization processes. The increasing complexity of the GRK2 "interactome" implies this kinase in several cardiovascular, inflammatory or tumour pathologies [92-94]

Using the crystallized structure of the GRK2-G*a*q-G*bg *complex as initial template (Figure 3A), and homology modelling procedures, a model was generated illustrating the putative interaction between Arg214 in the G*a*q chain and a putative phosphorylated Ser121 in the GRK2 chain (Figure 3B). As expected, the main qualitative changes in surface electrostatic properties correspond to an increase in the surface electro-negativity caused by the presence of an extra phosphate group in pSer212. This added negative charge complements the positive charge of Arg214, stabilizing the protein contact (Figure 3C). To obtain a quantitative comparison between both phospho- and unphosphorylated states of Ser121, a simulated molecular dynamics procedure was applied for 10 nanoseconds. The variation in the interaction complex was evaluated by continuous measuring of root-mean square deviation (rmsd) values with respect to the initial crystallized structure. The result, shown in Figure 3D, indicates that the presence of a phosphate group associated to Ser121 results in more stable interaction.

From a clinical perspective, this result would indicate that the presence of a mutated Ser121 residue in GRK2 will produce different effects depending on the nature of the new residue. A conservative mutation (*e.g*. S121A) will not cause important changes in the overall 3D structure of GRK2, but a consolidation of the "unphosphorylated" state, thus disturbing the protein-protein contact at this level. However, putative mutations such as S121D or S121E would generate a "constitutively phosphorylated-like state", stabilizing a reinforced interaction between the two polypeptides.

All these results can be also extrapolated to all members of the same family of proteins. Sequence analysis reveals high similarity values, indicative of close homology. Structure in Figure 2 corresponds to the bovine GRK2 protein. Human close homologues are: GRK2, GRK6, GRK5, GRK4 and GRK7. Sequence similarity between these proteins will allow comparative studies of the putative effect of Ser/Thr phosphorylation in the interaction of all these kinases with their respective G proteins.

## Conclusions

Aberrant activation of kinase signalling pathways is commonly associated with several types of cancer. Recent developments in phosphoprotein/phosphopeptide enrichment strategies, quantitative mass spectrometry and bioinformatic tools have resulted in robust pipelines for high-throughput characterization of phosphorylation in a global fashion.

It is possible to profile site-specific phosphorylation events on thousands of proteins in a single experiment. Chemical proteomic strategies have been used to unravel targets of kinase inhibitors, which are otherwise difficult to characterize. This approach's potential is already being used to characterize signalling pathways that govern oncogenesis. We summarized various approaches used for the analysis of the phosphoproteome in general and protein kinases in particular, highlighting key cancer phosphoproteomic studies.

Different proteomic and bioinformatic strategies need to be combined to achieve good phosphopeptide quantitative-protein studies. From the point of view of the so-called "personalized medicine", bioinformatics studies of reversible phosphorylation in proteins will allow the generation of models for protein-protein contacts at the atomic level taking into account each particular protein sequence. Molecular dynamic analysis of those contacts, be it in healthy people or in cancer studies, will allow the modification of the 3D computer models obtaining virtual structures tailored to individual patients. The next step in the future of drug development will be the generation of drugs specifically designed to each particular patient. It is necessary that clinicians, proteomics and bioinformatics work together in order to improve therapies and drug candidates development.

## List of Abbreviations

*Note: These abbreviations are useful proteomic abbreviations; some of them are mentioned and described in this Review, and they are also described in the References of this article*.

**AQUA: **Absolute Quantitation; **CID: **Collision-Induced Dissociation; **Da: **Dalton (molecular mass); **DIGE 2-D: **Fluorescence Difference Gel Electrophoresis; **ECD: **Electron Capture Dissociation; **ESI: **Electron Spray Ionization; **ETD: **Electron Transfer Dissociation; **FT-ICR: **Fourier transform-Ion Cyclotron Resonance; **HILIC: **Hydrophilic interaction chromatography; **HPLC: **High-performance liquid chromatography or high-pressure liquid chromatography; **H_3_PO_4 _**Phosphoric acid; **ICR: **Ion Cyclotron Resonance; **IMAC: **Immobilized Metal Affinity Capture; **IT: **Ion Trap; **iTRAQ: **Isobaric Tag for Relative and Absolute Quantitation; **kDa: **kilodalton (molecular mass); **LC: **Liquid Chromatography; **MALDI: **Matrix-Assisted Laser Desorption/Ionization; **MD: **Molecular Dynamics; **MOAC: **Metal Oxide Affinity Chromatography; **Mr: **Relative molecular mass (dimensionless); **MRM: **Multiple reaction monitoring; **MS: **Mass Spectrometry; **MSA: **MultiStage Activation; **MS/MS: **tandem mass spectrometry; **m/z: **Mass to charge ratio; **PID: **Primary Immunodeficiencies; **PTM: **Post-Translational Modification; **SILAC: **Stable Isotope Labelling with Amino acid in cell Culture; **SIMAC: **Sequential Elution from IMAC; **TiO_2 _**Titanium dioxide; **TOF: **Time Of Flight; **ZrO_2_**: Zirconium dioxide

## Competing interests

The authors declare that they have no competing interests.

## Authors' contributions

EL carried out the proteomics, phosphoproteomics and mass spectrometry studies for this review. JJW, JM and PGP carried out the bioinformatic studies for this review. IL and SMR carried out the clinical studies for this review. EL, JJW, IS, JM, PGP and SMR carried out these complementary studies in order to develop Clinical Phosphoproteomic-Bioinformatic research and publish this article. All authors read and approved the final manuscript.
